# A Five-Year Review of Perforated Peptic Ulcer Disease in Irrua, Nigeria

**DOI:** 10.1155/2017/8375398

**Published:** 2017-06-01

**Authors:** A. E. Dongo, O. Uhunmwagho, E. B. Kesieme, S. U. Eluehike, E. F. Alufohai

**Affiliations:** ^1^Department of Surgery, Irrua Specialist Teaching Hospital, Irrua, Nigeria; ^2^Ambrose Alli University, Ekpoma, Nigeria; ^3^Department of Radiology, Irrua Specialist Teaching Hospital, Irrua, Nigeria

## Abstract

**Background:**

Peptic ulcer perforation is a common cause of emergency admission and surgery. This is the first study that documents the presentation and outcome of management in Irrua, Nigeria.

**Patients and Method:**

This is a prospective study of all patients operated on for perforated peptic ulcer between April 1, 2010, and March 31, 2015. A structured questionnaire containing patients' demographics, operation findings, and outcome was filled upon discharge or death.

**Results:**

There were 104 patients. 81 males and 23 females (M : F = 3.5 : 1). The age range was between 17 years and 95 years. The mean age was 48.99 years ± SD 16.1 years. The ratio of gastric to duodenal perforation was 1.88 : 1. Perforation was the first sign of peptic ulcer disease in 62 (59.6%). Pneumoperitoneum was detectable with plain radiographs in 95 (91%) patients. 72 (69.2%) had Graham's Omentopexy. Death rate was 17.3%.

**Conclusion:**

We note that gastric perforation is a far commoner disease in our environment. Perforation is often the first sign of peptic ulcer disease. We identify fasting amongst Christians as a risk factor for perforation.

## 1. Introduction

Peptic ulcer perforation is a life threatening complication of peptic ulcer disease occurring in about 2–14% of cases of peptic ulcer disease [1, 2]. This perforation is either located in the lesser curvature of the stomach or on the anterior surface of the duodenum [3] resulting in a spillage of gastric contents into the peritoneal cavity. Perforation is one of the commonest causes of emergency hospitalization and surgery in peptic ulcer disease [4, 5].

The first clinical description of a perforated peptic ulcer was made in 1670 in princess Henrietta of England [6]. Since then several notable people have succumbed to this illness over the years [7]. The presentation may be dramatic with pain of sudden onset often severe and radiating to the back with rapidly supervening features of peritonitis in about two-thirds of patients [8]. In this classical presentation the patient may recall the exact time of perforation, often in the early hours of the morning. Pain may sometimes be insidious in onset and sometimes mimic an acute appendicitis [9] when perforation is small and contents leak slowly into the right iliac fossa through the right paracolic gutter [3]. In elderly patients, or immunocompromised patients, the signs of perforation may be insidious or equivocal [10].

The diagnosis is made with a high index of suspicion with the main differential being an acute exacerbation in a patient with known peptic ulcer disease [11]. The presence of air under the diaphragm in an erect chest radiograph often clinches the diagnosis. This sign, present in up to 75% [12] of erect chest radiographs, is dependent on size of perforation and interval before presentation. The use of an erect lateral chest radiograph can improve detection of pneumoperitoneum to 98% [13]. Currently, the use of computerized tomographic scan is the gold standard for detection of perforation [14, 15]. With ultrasonography, though easily accessible, and useful when radiation burden is critical [16], detection of pneumoperitoneum is difficult even for the skilled sonographer [17].

The aim of treatment is surgery after active resuscitation [18]. Few recent studies advocate nonoperative intervention except as a stop gap before definitive surgical intervention [11]. Recently, laparoscopic repair is being advocated when the expertise and equipment are available. Although outcome with open surgery is comparable [19], laparoscopic repair has the distinct advantage of reduced hospital stay as well as reduced postoperative pain and opiate requirement [20].

Nevertheless, in a resource-poor environment like ours, open surgery remains the only available option with either a simple closure or the use of an omental (graham's) patch [21] or champagne cork closure [22]. Because of our improved understanding of the pathogenesis of ulcers especially the role of* Helicobacter pylori*, the question of definitive antiulcer surgery at the same setting has few remaining indications [23–25]. When indicated [26], a careful evaluation of several factors like the presence of comorbidities, age, and the physiological state of the patient is required to improve mortality.

This study attempts to highlight the pattern of presentation and to document the outcome after surgical intervention in patients with perforated peptic ulcer disease in a rural community in mid-western Nigeria.

## 2. Patients and Method

This is a prospective study of all patients who had operative intervention for perforated peptic ulcers at the Irrua specialist teaching hospital over a 5-year period between April 1st, 2010, and March 31, 2015. Approval was sought and received from the ethics and research committee of the hospital before commencement of the study.

Irrua specialist teaching hospital is a 375-bedded hospital in Irrua, a rural community in mid-west Nigeria. It is about 100 kilometres from the state capital city of Benin. It serves principally the central and northern senatorial districts of Edo state and the neighbouring states of Ondo, Kogi, and Delta states. This population is about 3-4 million.

A questionnaire was filled by one of the authors or his residents within 3 days of surgery and upon discharge or death. Data collected include patient demographics, site and size of perforation, amount of pyoperitoneum interval before presentation, and type of surgery performed as well as treatment and outcome.

The diagnosis of perforated peptic ulcer was made on clinical grounds. This was confirmed at laparotomy. Patients were resuscitated with intravenous fluids and had baseline biochemical and hematological investigations done. Erect chest or lateral decubitus radiographs and abdominal ultrasound were carried out. No patient had computerized tomographic scan done as it was unavailable here during the period under study. All patients were catheterized and had nasogastric suction. Surgery was performed via a midline supraumbilical incision after adequate resuscitation. Simple closure or omentopexy was carried out with copious saline peritoneal lavage. The ulcer edge was excised for histology routinely. A drain was usually left in Morrison's pouch. All patients received triple regime antibiotics for 14 days for* H. pylori* eradication. Data were analyzed using SPSS 22 Statistical Package.

## 3. Results

**Figure 1:**
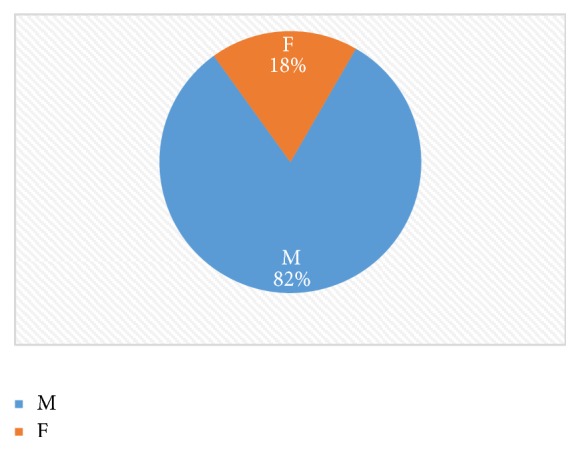
Pie chart showing gender distribution.

In the period under study, 104 patients had operative intervention for perforated peptic ulcer disease. There were eighty-one (81) males and twenty-three (23) females, giving a male to female ratio of 3.5 : 1 (Figure 1). Sixty-eight (65%) patients had perforated gastric ulcer while thirty-six (35%) patients had perforated duodenal ulcer giving a gastric to duodenal ulcer ratio of 1.88 : 1. All patients had a single perforation.

**Figure 2:**
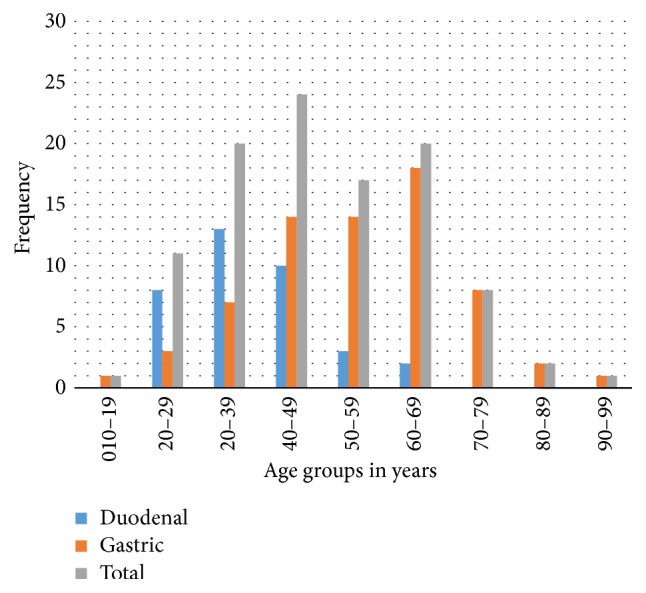
Bar chart showing age distribution and site of perforation.

The age range was between 17 years and 95 years (Figure 2). The mean age was 49.99 years with a standard deviation of 16.1 years. The mean age for the duodenal ulcer perforation was 37.75 years (SD 11.08 years). The mean age for gastric ulcer perforation was 55 years (SD 15.19 years).

A majority of patients, sixty-two (59.6%), had no history of peptic ulcer disease and only forty-five patients (43.2%) had admitted to taking any form of antiulcer medication within the last six months before perforation. Majority of the patients were from the lower socioeconomic groups. Farmers constituted the single largest group 41 (39.4%); traders were 12 (11.5%); students were 8 (7.7%); pastors and teachers were 6 each (5.7%) (Table 1).

The commonest mode of presentation was pain occurring in all the 104 patients (Table 2). The next commonest symptom was vomiting in 70 (67%) patients. Fever occurred in only 32 (31%) patients. Air under the diaphragm was found in 95 (91%) patients from plain chest or erect lateral decubitus radiographs. Risk factors identified include NSAID use in 39 (37.5%), including the youngest patient, ingestion of herbal concoctions in 18 (17.3%), dry fasting in 6 (5.7%), four pastors and two females, and smoking in 5 (4.8%).

The sizes of perforation ranged in <1 cm, 51 (49%); between 1 and 2 cm, 39 (37.5%); and >2 cm, 14 (13.5%). The quantity of pyoperitoneum at laparotomy ranged between (in litres) <1 L, 24 (23.1%); 1 and 2 L, 57 (54.8%), and >2 L, 23 (22.1%). The preferred method of repair was graham's omentopexy in 72 (69.2%) patients (see Table 3). The rest had simple closure of the edges. No patient had definitive antiulcer surgery. There were 9 reoperations, 4 for leakage of repair and 5 for intraabdominal collections with repair intact. None of the samples sent for histology revealed any malignancy.

Eighty-six patients (82.7%) were discharged home and there were 18 (17.3%) deaths in all. Two of the deaths had reoperations.

## 4. Discussion

In this study, a total of one hundred and six patients were operated on for gastroduodenal perforations. This gives an average of almost twenty-one cases annually. This figure is slightly higher in incidence than those described in Enugu Nigeria and some Eastern and Southern African series [27–29]. It is to be expected that this may be an underrepresentation as several late cases may have succumbed to the disease before definitive surgery and are thus not captured.

We find that peptic ulcer perforation is predominantly a male affliction as males outnumbered females by a ratio of 3.5 to 1. This finding is consistent with several others from Africa which confirm a male preponderance from a low 1.3 : 1 in Bugando, Tanzania [27], to a high ratio of 8.3 : 1 in Techiman, Ghana [22], and 14 : 1 in Ido Ekiti, Nigeria [30]. It is contrary to the common depiction in western series as a disease of the elderly female [31, 32].

In addition to the foregoing, there is the finding that peptic ulcer perforations affect a younger age group. The mean age for duodenal perforation is 37.75, almost 20 years lower than for gastric perforations. More than 75% of all duodenal perforations occur before the age of 50 years. It is to be noted however that the youngest patient in this series had a gastric perforation while taking ibuprofen for 1 week for severe low back ache from farm work. This drug has been implicated in peptic ulceration even in the paediatric age group previously [33].

Unlike previous studies from Nigeria which reveal no cases of gastric ulcer perforation [27, 30, 34], we now report gastric ulcers outnumbering duodenal perforation by a ratio of about 2 : 1. Although a previous study from a municipal hospital in Ghana had shown a similar finding [22], several other African studies reveal a majority of duodenal perforation [28, 35, 36]. While we have no clear explanation for this changing epidemiological profile, we note that gastroduodenal ulcers share similar pathogenesis especially of* H. pylori* infestation [37] which is commoner in younger patients in the lower socioeconomic rung [38]. Studies from Southern and Northern Nigeria confirm a high prevalence of 81.4% when using urease culture tests for antral biopsies and as high as over 90% with serological tests amongst dyspeptic patients [39, 40]. Although testing for* H. pylori* was unavailable in our centre during this study, all patients with perforated ulcers received eradication therapy for* H. pylori*. It is also known that abuse of nonsteroidal anti-inflammatory agents which we found in as high as 37.5% is a major etiologic agent especially in gastric ulceration. Other risk factors identified include use of herbal remedies previously alluded to by other workers and “dry” fasting. Dry fasting is described as fasting without drinking water or eating.

This study has found that perforation may be the first symptom of peptic ulcer disease since as much as three out of every five patients had no previous dyspeptic symptoms. It had been highlighted previously that diagnosis of peptic ulcer disease is only made after perforation in many developing countries [41]; only 43% of patients had admitted to taking any form of antiulcer medications in the 6 months preceding perforation. This figure is slightly higher than the 31% reported in Enugu, Nigeria, of perforations in patients known to have chronic peptic ulcer disease [27]. This finding has the distinct advantage of increasing the index of suspicion of perforation compared to acute exacerbation of peptic ulcer which may delay definitive surgical intervention. With perforation, however, we note that pain was universal in our series occurring in 100% of cases followed by vomiting in 71%. This finding is similar to Chalya et al. [28] who observed these two leading presenting features. Fever is a far less prevalent symptom in our patients occurring in only 31% of our patients. This finding may be due to the use of analgesics with antipyretic properties.

In the period under study, our centre had no computerized tomographic scan; inspite of this, this study shows a high detection rate of pneumoperitoneum of 91%. This is higher than what other studies suggest [12, 28] but similar to that found in Ghana [22, 36]. Late presentation may play a role as radiographic detection of pneumoperitoneum improves when interval between perforation and radiologic examination is long [42]. While multidetector CT has the distinct advantage of providing direct evidence of site gastrointestinal discontinuity [43] assisting in determining the best surgical option preoperatively in perforated peptic ulcers [44] we contend that, in our setting, plain radiographs are sufficient in the emergency patient with sudden onset epigastric pain as some workers have suggested [45].

In a rural community such as ours, understandably, majority of our patients would be in the lower socioeconomic groups. But this study identifies another risk group, clergymen or pastors. Four pastors over a five-year period were operated upon for perforated peptic ulcers. They all had gastric perforation and were all males. They were in the midst of dry fasting for between 3 and 7 days before they perforated. Two others, females one with duodenal and another with gastric perforation, were also admitted with symptoms while on a fast. Several studies in the past have documented the increased frequency of peptic ulcer and its complications during Ramadan fast [46–48]. Unlike the partial hunger that exists during Ramadan, a dry fast is likely to produce a higher frequency of complications within a shorter time frame from onset of fasting.

This study has shown that a repair with an omental patch or simple repair produces acceptable results even for ulcers that are relatively large as 13.5% of our patients had ulcers larger than 2 cm in diameter. Of the 9 patients who had reoperations after the procedure, 5 were found to have an intact repair at subsequent surgery. Two patients had fibrosis around the ulcer margin at the initial surgery and despite excision of the ulcer edges and a pedicled omental patch there was a leakage.

The overall mortality in our series of 17.3% is within the range 4–30% widely quoted in many series [49–51]. Two of our patients died after reoperations. Two died from pulmonary embolism. The rest from septicaemia, adult respiratory distress syndrome, and multiple organ failure.

In conclusion we note that perforated peptic ulcer is a common surgical problem in our environment. A majority of such perforations are gastric in nature and such perforations are the first sign of peptic ulcer disease in a majority of the patients. A plain chest radiograph is sufficient to make the diagnosis in the classic case of sudden onset epigastric pain. We identify fasting as an emerging risk factor for perforation amongst Christians.

## Figures and Tables

**Table 1:** Occupation.

Occupation	Number of patients	Frequency/percentage
Farmers	45	43.2
Traders	9	8.6
Students	7	6.7
Pastors	7	6.7
Teachers	5	4.8
Others	31	29.8

Total	104	99.8

**Table 2:** Clinical presentation and their frequency rate.

Clinical presentation	Frequency	Percentage
Pain	104	100
Vomiting	70	67
Fever	32	31
Constipation	24	23.2
Air under diaphragm	95	91
Abdominal distention	64	61.5

**Table 3:** Method of repair and frequency rate.

Repair method	Frequency (%)
Simple closure	32 (30.8)
Omental patch repair	72 (69.2)
